# Atovaquone Targets Mitochondrial Metabolism and Enhances Radiosensitivity of Diffuse Intrinsic Pontine Glioma

**DOI:** 10.3390/cancers18101553

**Published:** 2026-05-11

**Authors:** Faiqa Mudassar, Kristina M. Cook, Zachary N. Warnken, Neha Bal, Joey Lai, Brian Gloss, Holly P. McEwen, Ryan J. Duchatel, Geraldine M. O’Neill, Harriet Gee, Han Shen, Eric Hau

**Affiliations:** 1 Translational Radiation Biology and Oncology Laboratory, Centre for Cancer Research, The Westmead Institute for Medical Research, Sydney, NSW 2145, Australia; faiqa.mudassar@wimr.org.au (F.M.); 2 Faculty of Medicine and Health, Sydney Medical School, The University of Sydney, Sydney, NSW 2006, Australia; 3 Charles Perkins Centre, The University of Sydney, Sydney, NSW 2006, Australia; 4 Via Therapeutics LLC, Austin, TX 78712-0126, USA; 5 Westmead Research Hub Core Facilities, The Westmead Institute for Medical Research, Sydney, NSW 2145, Australia; 6 Cancer Signalling Research Group, School of Biomedical Sciences and Pharmacy, College of Health, Medicine and Wellbeing, University of Newcastle, Newcastle, NSW 2308, Australia; 7 Precision Medicine Research Program, Hunter Medical Research Institute, Newcastle, NSW 2305, Australia; 8 Children’s Cancer Research Unit, The Children’s Hospital at Westmead, Sydney, NSW 2145, Australia; 9 Western Sydney Radiation Oncology Network, Sydney, NSW 2145, Australia; 10 Children’s Medical Research Institute, Sydney, NSW 2145, Australia

**Keywords:** diffuse intrinsic pontine glioma, oxygen consumption rate, mitochondrial inhibition, radiation

## Abstract

Diffuse intrinsic pontine glioma (DIPG) is a lethal childhood brain tumor. Radiotherapy remains the standard of care, but tumors recur due to radioresistance. Tumor hypoxia contributes to radioresistance, and evidence of oxidative metabolism and hypoxia-associated transcriptomic programs suggests that hypoxia may be relevant in DIPG. We therefore investigated the FDA-approved mitochondrial inhibitor atovaquone as a strategy to target oxidative metabolism and enhance radiation response in DIPG. **Methods:** Patient-derived DIPG cell lines were used to evaluate atovaquone by extracellular flux analysis, hypoxia and reactive oxygen species assays, clonogenic survival assays, metabolomics, and RNA sequencing. To improve brain exposure, an amorphous solid dispersion (ASD) atovaquone formulation was evaluated and tested in an orthotopic DIPG model. **Results**: In patient-derived DIPG cultures, atovaquone suppressed mitochondrial respiration, reduced hypoxia-associated readouts, decreased HIF-1α expression in 3D models, and enhanced radiation response. At higher concentrations, atovaquone also increased oxidative stress and enhanced the radiosensitivity of DIPG monolayers. Transcriptomics analysis revealed disruption of cell-cycle and mitotic pathways, supporting additional treatment-associated effects beyond hypoxia reduction alone. Commercial and ASD formulations showed comparable in vitro activity. In vivo, ASD atovaquone combined with radiation prolonged survival in an orthotopic DIPG model. **Conclusions:** Targeting mitochondrial metabolism enhances radiosensitivity in DIPG and supports mitochondrial metabolism as a potential therapeutic weakness in this disease. Its effects are associated with reduced hypoxia-related signaling and broader metabolic and transcriptional changes.

## Simple Summary

Diffuse intrinsic pontine glioma (DIPG) is a fatal pediatric brain tumor in which radiotherapy remains the main treatment, but its benefit is limited due to radioresistance. One factor that may contribute to this resistance is the presence of low oxygen (hypoxia) within the tumor. In this study, we tested atovaquone, an FDA-approved drug that blocks mitochondrial energy production, as a way to improve the response of DIPG to radiation. We found that atovaquone reduced oxygen consumption in tumor cells, lowered markers associated with low oxygen, and improved radiation response in laboratory models. We also found evidence that oxidative stress and disruption of cell-cycle pathways may contribute to these effects. An improved formulation of atovaquone that increased brain exposure also prolonged survival in a mouse model when combined with radiation. These findings support further investigation of atovaquone and inhibition of mitochondrial metabolism as a possible radiosensitizing strategy for DIPG.

## 1. Introduction

Diffuse intrinsic pontine glioma (DIPG), a subset of diffuse midline glioma (DMG), is a fatal pediatric brainstem tumor with a median overall survival of under one year [1]. Radiotherapy remains the standard of care; however, tumor recurrence is inevitable in part because of radioresistance [2]. Hypoxia is common in solid tumors and contributes to radioresistance [3]. Low oxygen levels limit fixation of radiation-induced DNA damage, thereby reducing the effectiveness of radiotherapy [4]. Hypoxia-inducible factors (HIFs) can also promote radioresistance and have been implicated in the pathogenesis of pediatric high-grade glioma (HGG) [5]. Reports of tissue hypoperfusion in DIPG patients suggest a hypoxic tumor microenvironment may be present [6]. Our recent work further identified hypoxia-related transcriptional signatures in clinical DIPG samples and elevated hypoxia markers in DIPG mouse models, supporting this premise [7]. Targeting tumor hypoxia may therefore enhance the efficacy of radiotherapy in DIPG.

Attempts to enhance oxygen delivery to tumors have achieved limited clinical success [8,9,10]. An alternative is to reduce tumor oxygen demand by inhibiting mitochondrial oxidative phosphorylation (OXPHOS), thereby limiting cellular oxygen consumption [8,11]. OXPHOS is upregulated in several cancers [12] and OXPHOS inhibition alleviates hypoxia and enhances radiosensitivity in several preclinical models [13,14,15]. DIPG also exhibits upregulated OXPHOS metabolism in vitro [7,16]. Our previous study demonstrated that the OXPHOS inhibitor phenformin reduced hypoxia in vitro and improved survival in an orthotopic DIPG mouse model when combined with radiation [7]. However, phenformin’s risk of lactic acidosis and market withdrawal highlight the need for safer OXPHOS inhibitors to enhance radiotherapy response.

The FDA-approved anti-malarial drug atovaquone is an OXPHOS inhibitor with anti-cancer potential. As a ubiquinone analog, it inhibits the ubiquinol oxidation (Q_o_) site of mitochondrial complex III [17]. Atovaquone reduced oxygen consumption rate (OCR) and hypoxia in several cancer models and improved survival in a hypopharyngeal carcinoma xenograft with radiation [14]. Its hypoxia-reducing effect has been confirmed clinically [18], leading to ongoing trials combining it with chemoradiotherapy in lung cancer (NCT04648033). However, its efficacy in brain tumors, including DIPG, remains unstudied. Given its limited blood-brain barrier (BBB) penetration [19], improved formulations will be needed to achieve therapeutic brain concentrations.

Here, we investigated the potential of atovaquone to enhance radiotherapy response in DIPG through inhibition of mitochondrial OXPHOS. We further examined molecular pathways associated with treatment response using transcriptomic and metabolic profiling. Because brain delivery is a key barrier to clinical translation, we also evaluated an amorphous solid dispersion (ASD) formulation of atovaquone in an orthotopic DIPG model.

## 2. Materials and Methods

### 2.1. Cell Culture and Compounds

Primary patient-derived DIPG cell lines SU-DIPG-XVII and SU-DIPG-VI were gifted from the Monje Lab (Stanford University, Stanford, CA, USA), while HSJD-DIPG-007 was obtained from Sant Joan de Déu Research Foundation (Barcelona, Spain). These cell lines represent H3K27M-mutant DIPG models and have been previously characterized. HSJD-DIPG-007 and SU-DIPG-VI were cultured as neurospheres, whereas SU-DIPG-XVII was maintained as monolayers. The neurosphere models were used as they better recapitulate three-dimensional tumor architecture and oxygen gradients, whereas the monolayer model was used to assess treatment effects independent of hypoxia. DIPG cells were cultured in a 1:1 mixture of DMEM/F12 (Cat. no. 11320082, Thermo Fisher Scientific, Waltham, MA, USA) and Neurobasal-A media (Cat. no. 10888022, ThermoFisher Scientific) supplemented with 1× Non-Essential Amino Acids (Cat. no.11140050, ThermoFisher Scientific), 1× GlutaMAX-I Supplement (Cat. no. 35050061, ThermoFisher Scientific), 1 mM MEM Sodium Pyruvate Solution (Cat. no. 11360070, ThermoFisher Scientific), 10 mM HEPES buffer (Cat. no. 15630080, ThermoFisher Scientific), 1× Antibiotic-Antimycotic (Cat. no. 15240096, ThermoFisher Scientific), 1× B27 supplement (Cat. no. 12587001, ThermoFisher Scientific), 2 μg/mL Heparin (Cat. no. 7980, Stem Cell Technologies, Vancouver, BC, Canada), 10 ng/mL PDGF-AA (Cat. no. 100-16-100UG, Jomar Life Research, Mulgrave, VIC, Australia), 1 ng/mL PDGF-BB (Cat. no. 100-18-100UG, Jomar Life Research), 20 ng/mL human-EGF (Cat. no. 100-26-100UG, Jomar Life Research), 20 ng/mL human-FGF (Cat. no. 100-146-100UG, Jomar Life Research). The immortalized human astrocytes cell line (IHA-hTERT) was purchased from Resolving Images (Melbourne, VIC, Australia) and cultured in extracellular matrix-coated flasks using Prigrow IV medium (Cat. no. TM004, Resolving Images) supplemented with 10% FBS (Cat. no. TM999-100, Resolving Images) 10 ng/mL EGF, 1% L-glutamine (Cat. no. G275, Resolving Images) and 1% penicillin/streptomycin (Ca t. no. G255, Resolving Images). All the cell lines were maintained in humidified incubators at 37 °C and 5% CO_2_. The cell lines were authenticated by short tandem repeat (STR) profiling and routinely tested for mycoplasma contamination. Atovaquone (Cat. no. A7986) was purchased from Sigma-Aldrich (Sydney, NSW, Australia). ASD atovaquone formulation was kindly provided by Zachary N. Warnken (Via Therapeutics, LLC, Austin, TX, USA).

### 2.2. Cell Viability Assay

Cell viability was assessed using the MTS cell proliferation assay. SU-DIPG-XVII (3000 cells/well), SU-DIPG-VI (5000 cells/well), HSJD-DIPG-007 (3000 cells/well), and IHA-hTERT (4000 cells/well) were seeded in 96-well plates and treated with atovaquone for 24 h and 72 h. Following treatment, MTS reagent (Cat. No. G3581, Promega, Sydney, NSW, Australia) was added according to the manufacturer’s instructions. Absorbance was measured at 490 nm using the SpectraMax iD5 plate reader (Molecular Devices, San Jose, CA, USA).

### 2.3. Patient Cohorts

To evaluate the expression of mitochondrial electron transport chain (ETC) complex III genes in DIPG/DMG, patient mRNA data were obtained from the Pediatric cBioportal tool using the Open Pediatric Cancer (OpenPedCan) Project v15 dataset (downloaded on 28 April 2026) [20,21]. Expression data from non-tumor brain tissue were obtained from the GTEx database, including 246 caudate and 202 hypothalamus samples. Tumor samples comprised 10 brainstem glioma DIPG and 220 DMG-H3K27M samples. Genes encoding ETC complex III subunit (*CYC1*, *UQCRC1*, *UQCRC2*, *UQCRH*, *UQCRQ*, *ttc19*, *UQCRB*, *UQCRFS1*, *UQCR10*, *UQCR11*, *MT-CYB*) were analyzed following log2 transformation of mRNA expression data. Expression levels were compared between tumor and non-tumor brain tissues using one-way ANOVA with Tukey’s multiple comparisons test.

### 2.4. Extracellular Flux Assay

OCR and extracellular acidification rate (ECAR) were measured using Seahorse XF24 analyzer (Agilent Technologies, Santa Clara, CA, USA). SU-DIPG-XVII (40,000 cells/well), HSJD-DIPG-007 (65,000 cells/well), SU-DIPG-VI (100,000 cells/well), and hTERT IHA (80,000 cells/well) were treated with atovaquone for 4 h. OCR/ECAR were measured in XF Assay Medium (supplemented with 10 mM glucose, 1 mM sodium pyruvate, and 2 mM glutamine) according to standard Seahorse protocol (Agilent Technologies). The XF Cell Mito Stress Test (Cat. no. 103015-100, Agilent Technologies) was performed according to the manufacturer’s instructions. The cells were pretreated with atovaquone for 24 h prior to treatment with 1 μM oligomycin, 1 μM FCCP, and 0.5 μM rotenone/antimycin A.

For the mitochondrial complex-specific inhibition assay, HSJD-DIPG-007 cells were treated with 30 μM Atovaquone (4 h), 2 μM Antimycin A (Cat. no. A8674, Sigma-Aldrich) for 30 min, and 2 μM Myxothiazol (Cat. no. T5580, Sigma-Aldrich) for 30 min. Cells were then permeabilized using 3 nM XfPMP Reagent (Cat. no. 102504-100, Agilent Technologies) according to the manufacturer’s instructions. Complex-specific activity was determined by measuring changes in OCR in response to different injections. Seahorse media containing 10 mM pyruvate (Cat. no. 103578-100, Agilent Technologies), 1 mM malate (Cat. no. 02288, Sigma-Aldrich), and ADP (Cat. no. A5285, Sigma-Aldrich) was used and injections were as follows—injection 1 (2 μM rotenone, complex I inhibitor, cat. no. R8875, Sigma-Aldrich), injection 2 (10 mM succinate/2 μM rotenone, complex II substrate and complex I inhibitor: Cat. no. S3674, Sigma-Aldrich), injection 3 (2 μM Antimycin A, complex III inhibitor), injection 4 (10 mM ascorbate/100 μM TMPD, complex IV substrate; Cat no. A5960 and Cat. no. 87890, Sigma-Aldrich). Complex-specific OCR was calculated as follows: Complex I: Baseline OCR—OCR at rotenone injection; Complex II: OCR after succinate injection—OCR at rotenone injection; Complex III: OCR after succinate injection—OCR at antimycin A injection; Complex IV: OCR after ascorbate/TMPD injection—OCR at antimycin A injection.

To obtain complex III specific inhibition, the injections were -injection 1 (2 μM rotenone/10 mM succinate, complex I inhibitor and complex II substrate), injection 2 (10 mM malonate, complex II inhibitor; Cat. no. 360899, Sigma-Aldrich), injection 3 (0.5 mM duroquinol, complex III substrate; Cat. no. T0822, Chemsupply, Adelaide, SA, Australia), injection 4 (10 mM ascorbate/100 μM TMPD, complex IV substrate). Complex-specific OCR was calculated as follows: Complex II: OCR after succinate injection—Baseline OCR; Complex III: OCR after duroquinol injection—OCR at malonate injection.

### 2.5. Western Blot

DIPG cells were pretreated with atovaquone for 24 h. Cells were lysed in RIPA buffer with protease/phosphatase inhibitors and DNase I. Protein levels were quantified by Pierce BCA Protein Assay Kit as per manufacturer’s instructions. Proteins were separated by SDS-PAGE at 200 V for 32 min and transferred using the iBolt 2 Dry Blotting System. Antibodies include anti-HIF-1α (ab51608, 1:1000, Abcam, Adelaide, SA, Australia), anti-beta-actin (4967S, 1:1000, Cell Signaling Technology, Danvers, MA, USA) and HRP-linked anti-rabbit IgG (7074S, 1:2000, Cell Signaling Technology).

### 2.6. 3D Neurosphere Hypoxia Assay

HSJD-DIPG-007 (4000 cells/well) were seeded in 96-well ultra-low attachment U-bottom plates, incubated for 72 h to obtain neurospheres ∼500 μm in diameter prior to atovaquone treatment for 24 h. Neurospheres were stained with 5 μM Image-iT^TM^ Green Hypoxia Reagent (Cat. no. I14833, Thermo Fisher Scientific) and 1 μg/mL Hoechst 33,342 (Cat. no. 62249, ThermoFisher Scientific) for 1 h at 37 °C, then incubated in fresh medium for 3 h before imaging on Olympus FV1000 Confocal Microscope (Olympus Corporation, Tokyo, Japan) (488 nm, 405 nm).

### 2.7. Colony Formation Assay

For monolayer cells, a liquid colony formation assay with crystal violet staining was performed. SU-DIPG-XVII cells (grown as monolayers) or HSJD-DIPG-007 (seeded on laminin to grow as monolayers) were seeded at various seeding densities, treated with atovaquone for 6 h, followed by 2–4 Gy radiation delivered using Cell-Rad (Precision X-ray). After 24 h of total drug treatment, the media were refreshed, and colonies were allowed to form over 10–12 days before fixation and staining with 0.5% crystal violet (Cat. no. C0775-100G, Sigma-Aldrich) in 25% methanol. For suspension cells, a soft agar colony formation assay with Thiazolyl Blue Tetrazolium Bromide (MTT) (Cat. no. M2128, Sigma-Aldrich) staining was followed. Briefly, HSJD-DIPG-007 and SU-DIPG-VI cells (3 × 10^5^ cells/well, 6-well plates) were treated with atovaquone for 6 h, followed by 2–4 Gy radiation delivered using Cell-Rad (Precision X-ray). After 24 h of total drug treatment, cells were embedded in 0.3% agarose (Cat. no. 50100, Lonza Bioscience, Walkersville, MD, USA) over a 0.6% agarose base and cultured for 2 weeks. Colonies were stained with MTT (5 mg/mL) and quantified to calculate plating efficiency and surviving fraction.

### 2.8. Reactive Oxygen Species Assay

Mitochondrial and cytosolic ROS were assessed using MitoSOX™ Red (Cat. no. M36008, ThermoFisher Scientific) and DHE (Cat. no. D11347, ThermoFisher Scientific) dyes using flow cytometric assays. SU-DIPG-XVII and HSJD-DIPG-007 (3 × 10^5^ cells/well) were treated with atovaquone for 6 h ± 4 Gy radiation and collected 24 h post-drug (total drug time 24 h; radiation time 18 h). Mitochondrial and cytosolic ROS were assessed using MitoSOX™ Red (50 μM, 30 min) and DHE (2.5 μM, 30 min) at 37 °C. After washing with HBSS or PBS, Sytox Blue (1 μM, 5 min) was added. Samples were analyzed on a BD FACSCanto II (PE: excitation 488 nm emission 585/42 nm; Pacific Blue: excitation 405 nm emission 450/50 nm).

### 2.9. RNA Sequencing

RNA was extracted 24 h post-treatment using RNeasy mini handbook (Qiagen, Clayton, VIC, Australia). High-quality RNA (RIN > 8) was used for library preparation (Illumina Stranded mRNA kit) and sequenced on an Illumina NovaSeq 6000 (SP flow cell, single-end 100 bp reads). The detailed protocol is published in [7].

### 2.10. Metabolomics

HSJD-DIPG-007 cells were seeded in a 100 mm dish per condition with 5 replicates (2 × 10^6^ cells per dish). After 24 h incubation, cells were treated with atovaquone and/or radiation, followed by the extraction of metabolites. Cells were collected, washed with ice-cold PBS, and centrifuged at 750× *g* for 5 min at 4 °C. Pellets were lysed in 1 mL pre-chilled extraction buffer (1:1 Optima^®^ LC/MS Methanol:Water; Fisher Chemical, Cat. no. A456 and W6), followed by addition of 1 mL Chromasolv™ Chloroform (Cat. no. 366927, Honeywell Fluka, Charlotte, NC, USA). Samples were vortexed, incubated on ice for 10 min (with intermittent vortexing), and centrifuged at 15,000× *g* for 10 min at 4 °C. The aqueous phase was mixed with acetonitrile/methanol/formic acid (75:25:0.2, *v*/*v*/*v*) or acetonitrile/methanol (75:25, *v*/*v*) for HILIC separation using either an Atlantis^®^ HILIC column or an XBridge™ Amide column (Waters, Sydney, NSW, Australia). After a final centrifugation (15,000× *g*, 20 min, 4 °C), supernatants were injected into an LC-MS/MS platform comprising a Shimadzu Nexera LC-40B X3 UHPLC system (Shimadzu Corporation, Kyoto, Japan), equipped with a SIL-40C X3 autosampler and a CTO-40C column oven, coupled to an AB SCIEX QTRAP 6500+ mass spectrometer (SCIEX, Marlborough, MA, USA). Data were processed using MultiQuant SCIEX OS Software version 3.0.3, normalized to cell count or protein, log-transformed, and mean-centered using MetaboAnalyst 5.0 and GraphPad Prism. Volcano plots were generated with a fold change threshold of 1.1 and FDR-adjusted *p*-value < 0.1 (treatment vs. control).

### 2.11. Animal Studies

Animal experiments were approved by WSLHD-AEC (4370.10.22). HSJD-DIPG-007 (2 × 10^5^ cells in 2 μL Matrigel) were intracranially injected into the brainstem of female balb/c nude mice (6–8 weeks) using the KOPF Stereotactic Frame at coordinates 6.0 mm posterior to bregma, 0.5 mm lateral to midline, and at a depth of 3.5 mm. Mice were monitored daily and euthanized upon neurological signs or >20% weight loss.

To assess the efficacy of atovaquone in combination with radiation, mice were randomized into four groups (*n* = 8 per group): control, ASD atovaquone, radiation alone, or combination treatment. ASD atovaquone (200 mg/kg) was administered via oral gavage starting 21 days post-intracranial injection for 12 consecutive days. For combination treatment, ASD atovaquone was administered 1 h prior to radiation, with the final 5 days of atovaquone treatment coinciding with radiation treatment. Whole-brain radiation was delivered using X-RAD 320 irradiator (Precision X-ray, Madison, CT, USA) at a dose of 2 Gy per fraction for 5 days (total 10 Gy), starting on 28 days post-intracranial injection. Mice were restrained using individual fixtures, and lead shielding was used to cover the body, exposing only the head during irradiation. Mice were monitored daily following radiation for signs of distress or adverse effects. The control and radiation-alone groups were shared with our study in [7].

For the ASD atovaquone brain concentration analysis, mice received ASD atovaquone for 6 consecutive days. On day 6, brains were obtained, snap frozen, weighed, and then homogenized in ice-cold methanol using a FastPrep-24 5G lysis system (MP Biomedicals, Irvine, CA, USA). The brain sample was diluted using ice-cold methanol containing a spike of internal standard (Ponatinib to a final concentration of 1000 fmol/μL). For plasma samples, 20 μL was taken and diluted to 400 μL with ice-cold methanol containing a spike of internal standard (Ponatinib to a final concentration of 1000 fmol/μL). Two sets of matrix-matched calibration standards ranging from 10 fmol/μL to 5000 fmol/μL atovaquone were prepared using untreated mouse brain and plasma and containing 1000 fmol/μL of internal standard. All samples were shaken for 30 min at 1500 rpm at room temperature using an Eppendorf Thermoblock, then centrifuged at 18,000× *g* for 7 min. 200 μL of supernatant was transferred to a Mass Spectrometry (MS) vial for analysis. Reverse phase LC-MS/MS was performed using a Shimadzu analytical flow high-performance liquid chromatography system coupled to a SCIEX 6500 Qtrap mass spectrometer (SCIEX, Marlborough, MA, USA) with an electrospray ion source. 1 μL of each sample was separated on a Phenomenex Luna Omega 1.6 µm Polar C18 100 × 2.1 mm column, employing a gradient of 2–99% solvent B (solvent A = 0.1% formic acid, solvent B = acetonitrile, 0.1% formic acid) at a flow rate of 300 μL/min over 6 min. Multiple reaction monitoring (MRM) was performed in negative mode using optimized source parameters. Briefly, these were source gas: 40; ion spray voltage: −3500; source temperature: 450 °C. Entrance potential and delustering potential were optimized for atovaquone and equaled −8 and −165, respectively. The ion transition m/z 365.0 -> 337.0 (CE: −41) was used for quantification of atovaquone, and the additional transitions m/z 365.0 -> 227.1 (CE: −45) and 365.0 -> 171.1 (CE: −60) were monitored for qualitative peak validation. Peak integration was performed in Skyline, using internal standard abundance to normalize between samples. Atovaquone concentration was quantified by interpolation using the matrix-matched calibration curves.

### 2.12. Statistical Analysis

Statistical analyses were performed using GraphPad Prism 8. Data are presented as mean ± SD from at least two independent experiments. Significance was determined using Student’s *t*-tests or one-way ANOVA with Tukey’s multiple comparisons test, and survival by log-rank (Mantel-Cox) test. *p* < 0.05 was considered significant (* *p* < 0.05, ** *p* < 0.01, *** *p* < 0.001, **** *p* < 0.0001).

## 3. Results

### 3.1. Atovaquone Suppresses Oxidative Phosphorylation in DIPG Cells Without Early Cytotoxicity

**Figure 1:**
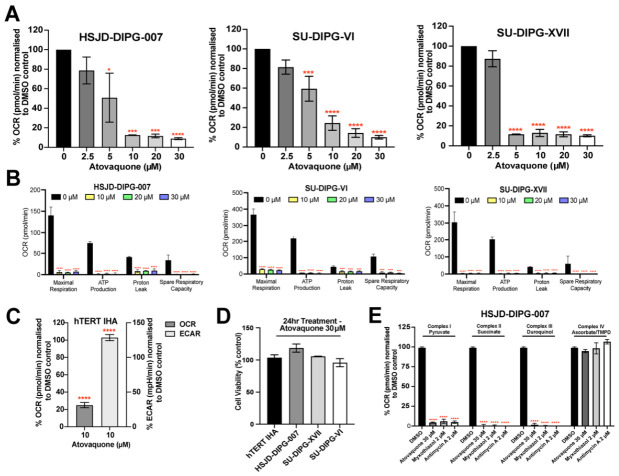
Atovaquone suppresses mitochondrial respiration in DIPG cells. (**A**) Acute atovaquone treatment reduces OCR in HSJD-DIPG-007, SU-DIPG-XVII, and SU-DIPG-VI cells, measured by Seahorse XFe24 analysis and normalized to DMSO control; (**B**) Seahorse XF Cell Mito Stress Test showing reduced maximal respiration, ATP-linked respiration, proton leak, and spare respiratory capacity following 24 h atovaquone treatment in DIPG cell lines; (**C**) Metabolic changes in OCR and ECAR of hTERT IHA following atovaquone treatment normalized to DMSO control. (**D**) Effect of atovaquone on the viability of DIPG cell lines and hTERT IHA assessed using MTS cell proliferation assay; (**E**) Effect of atovaquone, myxothiazol, and antimycin A on the complex I, II, III, and IV dependent OCR on HSJD-DIPG-007 cells. One-way ANOVA with Tukey’s multiple comparisons test was performed for all experiments (* *p* < 0.05, *** *p* < 0.001, **** *p* < 0.0001). Data are presented as means ± SD of two independent experiments with three replicates in each.

Given atovaquone’s known mitochondrial inhibition, we assessed its impact on the OCR and extracellular acidification (ECAR) in three DIPG cell lines, HSJD-DIPG-007, SU-DIPG-VI, and SU-DIPG-XVII. Acute treatment for 4 h reduced OCR in all lines in a dose-dependent manner, with a significant decrease at 5–30 μM, while ECAR was not significantly changed (Figure 1A and Figure S1). To define the broader effects on mitochondrial function, we next performed Seahorse Mito Stress assays following 24 h treatment. Atovaquone reduced maximal respiration, ATP-linked respiration, spare respiratory capacity, and proton leak across all three DIPG cultures (Figure 1B).

We then assessed the metabolic response on hTERT-IHA. 10 μM atovaquone reduced the OCR of hTERT-IHA within 4 h (Figure 1C). However, unlike in DIPG cells, atovaquone increased the ECAR in hTERT-IHA, suggesting a compensatory shift towards glycolysis (Figure 1C). To determine whether these metabolic effects reflected early cytotoxicity, we measured cell viability after atovaquone treatment. No significant reduction in cell viability was observed in any cell line following 24 h from the maximum atovaquone dose of 30 μM (Figure 1D), indicating that metabolic effects occurred independently of cytotoxicity. However, prolonged atovaquone exposure (72 h) resulted in a significant reduction in the viability of HSJD-DIPG-007 and SU-DIPG-XVII cells, whereas SU-DIPG-VI exhibited only a modest decrease in viability, comparable to that observed in hTERT-IHA (Figure S2).

### 3.2. Atovaquone Inhibits Mitochondrial Activity in DIPG Cells in a Manner Consistent with Mitochondrial Complex III Inhibition

Atovaquone inhibits complex III in other cancer models [14,22,23,24]; therefore, we assessed its impact on the activity of ETC complexes I-IV in DIPG cells. As shown in Figure 1E, treatment with 30 μM atovaquone significantly reduced the OCR of complexes I-III. This inhibition was not reversed by supplementation with complex-specific substrates—pyruvate (complex I), succinate (complex II), and duroquinol (complex III), indicating that provision of upstream or complex III-linked substrates was insufficient to restore electron flow. In contrast, supplementation with ascorbate/TMPD, which donates electrons to complex IV, restored OCR to control level (Figure 1E). To further understand this profile, atovaquone was also compared to the known complex III inhibitors, myxothiazol and antimycin A. There was a similar decrease in OCR of complexes I-III from myxothiazol and antimycin A, whereas complex IV was not impaired (Figure 1E). These findings support complex III as the principal site through which atovaquone inhibits mitochondrial respiration in DIPG cells.

We next examined mitochondrial complex III gene expression in DIPG/DMG samples, given that atovaquone is reported to target this complex. mRNA expression data were obtained from the Open Pediatric Cancer (OpenPedCan) Project v15 via cBioportal. Brainstem DIPG and DMG-H3K27M were compared with non-tumor brain tissues—caudate and hypothalamus from GTEx. Analysis of all complex III subunit genes revealed heterogeneous expression patterns across DIPG/DMG samples, with some genes showing increased expression and others reduced expression relative to non-tumor controls (Figure S3).

### 3.3. Atovaquone Reduces Hypoxia and HIF-1α and Enhances Radiosensitivity in DIPG Neurospheres

**Figure 2:**
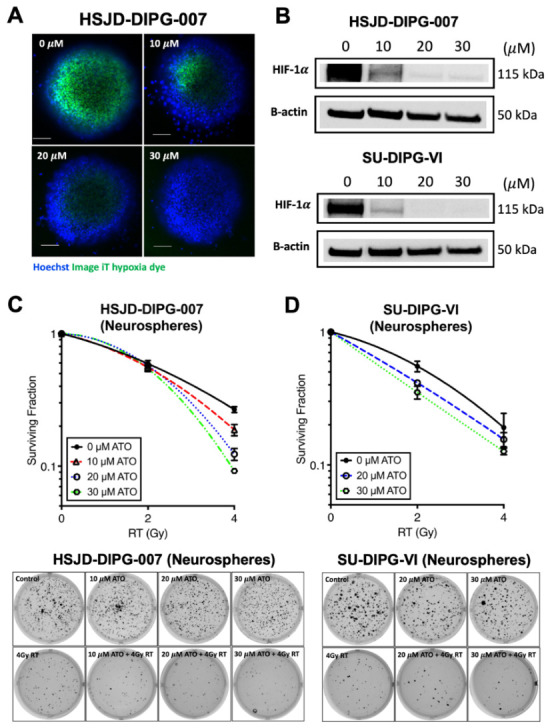
Atovaquone reduces hypoxia, HIF-1α, and radiosensitizes DIPG neurosphere models. (**A**) Atovaquone reduces hypoxia in HSJD-DIPG-007 neurospheres, measured using image iT^TM^ green hypoxia dye (green fluorescence). Blue fluorescence indicates nuclei staining by Hoechst dye. Five replicates per condition were set up, and the experiment was performed twice. Scale bar = 100 μm; (**B**) Effect of atovaquone treatment on HIF-1α expression in HSJD-DIPG-007 and SU-DIPG-VI neurospheres. Both the neurosphere models were cultured for 3 days prior to atovaquone treatment; (**C**,**D**) Effect of atovaquone on radiation-induced clonogenic cell death using soft agar assays. Surviving fraction graph (left) and representative colony formation images (right) are shown. ATO, atovaquone; RT, radiation. Original western blots are presented in File S1.

Because inhibition of oxidative metabolism is expected to reduce cellular oxygen consumption, we next tested whether atovaquone altered the presence of hypoxia in HSJD-DIPG-007 neurospheres. Neurospheres form hypoxic cores when reaching ~200 μm in diameter [25], providing a relevant in vitro model to assess this effect. Hypoxia was visualized using the image-iT^TM^ green hypoxia reagent, which is nonfluorescent in cells under normal oxygen concentrations and emits fluorescence when oxygen levels reach below 5%. Treatment with atovaquone decreased hypoxia staining with a near-complete loss of signal seen at 30 μM (Figure 2A). To further assess the hypoxic response, we measured HIF-1α in neurospheres. Atovaquone treatment reduced HIF-1α expression, with a complete loss observed at 20–30 μM in both neurosphere DIPG models HSJD-DIPG-007 and SU-DIPG-VI (Figure 2B). We then evaluated the radiosensitizing effect of atovaquone in neurosphere models using a soft agar assay. HSJD-DIPG-007 and SU-DIPG-VI neurospheres were pretreated with atovaquone, followed by radiation exposure. Atovaquone enhanced radiosensitivity in both DIPG cultures, with greater sensitivity observed in combination treatment compared to control and single treatments (Figure 2C,D). These findings suggest that atovaquone reduces hypoxia-associated signaling in 3D DIPG models and enhances their radiosensitivity in vitro.

### 3.4. Atovaquone Increases Oxidative Stress and Enhances Radiosensitivity in DIPG Monolayers

**Figure 3:**
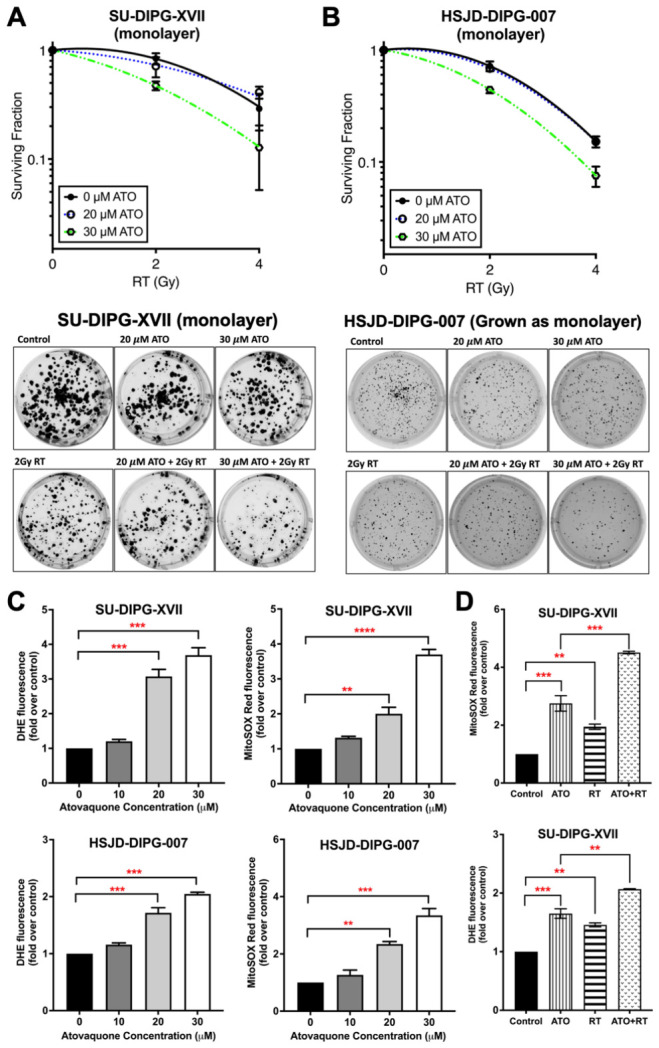
Atovaquone increases oxidative stress and radiosensitizes DIPG monolayer cultures. (**A**) Effect of atovaquone on radiation-induced clonogenic cell death in SU-DIPG-XVII monolayer cells; (**B**) Effect of atovaquone on radiation-induced clonogenic cell death in HSJD-DIPG-007 cells cultured as monolayers. Surviving fraction curves (left) and representative colony formation images (right) are shown for A and B. (**C**) DHE and MitoSOX Red fluorescence, normalized to untreated control, in SU-DIPG-XVII and HSJD-DIPG-007 cells, treated with 10–30 μM atovaquone, as analyzed using flow cytometry; (**D**) DHE and MitoSOX Red fluorescence, normalized to untreated control, in SU-DIPG-XVII cells treated with 30 μM atovaquone followed by 4 Gy RT as analyzed using flow cytometry. One-way ANOVA with Tukey’s multiple comparisons test was performed for all experiments (** *p* < 0.01, *** *p* < 0.001, **** *p* < 0.0001). Data are presented as means ± SD of two independent experiments with three replicates in each. ATO, atovaquone; RT, radiation.

We next assessed whether atovaquone altered radiation response in DIPG monolayers, which do not recapitulate the hypoxic gradients present in neurosphere cultures. In clonogenic survival assays, both SU-DIPG-XVII and HSJD-DIPG-007 cells grown under monolayer conditions, radiosensitization was evident only at 30 μM atovaquone (Figure 3A,B), suggesting that at higher concentrations, atovaquone may engage mechanisms beyond hypoxia modification alone. We next examined oxidative stress as a potential contributor to this effect. Atovaquone increased mitochondrial and cellular ROS in both cell lines, as shown by increased MitoSOX Red and DHE fluorescence (Figure 3C). In SU-DIPG-XVII cells, the combination of 30 μM atovaquone and 4 Gy radiation further increased both ROS readouts compared with either treatment alone (Figure 3D). These data support a model in which atovaquone-induced oxidative stress contributes to enhanced radiation response in DIPG monolayers.

### 3.5. Metabolomics and Transcriptomics Reveal Broad Metabolic Disruption and Altered Stress and Cell Cycle Transcription Programs Following Atovaquone ± Radiation Treatment

**Figure 4:**
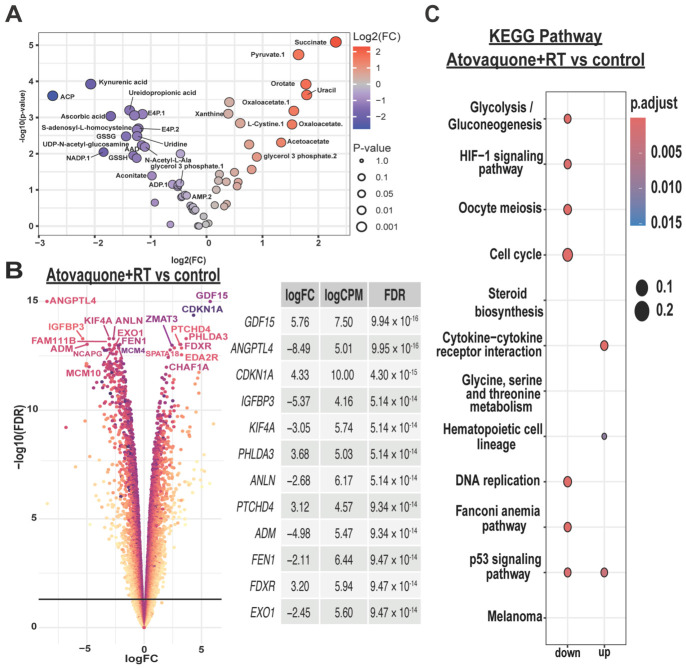
Multi-omics profiling identifies metabolic disruption and altered stress and cell-cycle transcriptomic programs following atovaquone treatment in HSJD-DIPG-007 cells. (**A**) Volcano plot of metabolites changes in HSJD-DIPG-007 cells treated with atovaquone compared to control; (**B**) Volcano plot of differentially expressed genes in HSJD-DIPG-007 cells treated with atovaquone + radiation versus control. Selected genes with the largest fold changes are labelled, and the adjacent table lists the top 12 differentially expressed genes ranked by fold change (FDR < 0.05). (**C**) KEGG pathway enrichment analysis for atovaquone plus radiation versus control. Dot size represents gene ratio, and color indicates adjusted *p* value; pathways are shown according to direction of enrichment (up- or down-regulated).

To explore whether atovaquone-associated radiosensitisation was accompanied by broader molecular reprogramming beyond hypoxia reduction and ROS induction, we performed transcriptomic and metabolomic profiling of HSJD-DIPG-007 cells following treatment with atovaquone and/or radiation. Metabolite levels were quantified using liquid chromatography-tandem mass spectrometry (LC-MS/MS). Principal component analysis (PCA) of the metabolomics data showed clustering of atovaquone and atovaquone + radiation groups, both of which were clearly separated from control and radiation alone (Figure S4), suggesting that atovaquone exerts the dominant effect on metabolism. Atovaquone treatment resulted in marked accumulation of succinate, pyruvate, and oxaloacetate, together with reduced aconitate (Figure 4A). These shifts are consistent with impaired mitochondrial oxidative metabolism and disrupted TCA cycling, supporting broader metabolic reprogramming.

We next examined transcriptional changes associated with treatment response. RNA sequencing revealed broad gene expression changes following atovaquone with or without radiation, with the top 50 upregulated and downregulated genes listed in Tables S2 and S3. Among genes selectively altered in the combination group, the most strongly downregulated transcripts included key regulators of DNA replication and repair (*MCM4*, *MCM10*, *TICRR*, *FEN1*, *EXO1*), mitotic progression (*KIF15*, *NCAPH*, *ANLN*), and chromatin assembly (*CHAF1A*, *WDR76*) (Table 1; Figure S5). The top DEGs unique to atovaquone alone are presented in Table S1 and Figure S6.

Consistent with these findings, volcano plot analysis of the combination group highlighted upregulation of stress-associated genes, including *GDF15* and *CDKN1A*, together with downregulation of genes involved in mitotic regulation and DNA replication, including *KIF4A*, *ANLN*, *EXO1*, *MCM10*, and *FEN1* (Figure 4B). Overall, these transcriptional changes are consistent with activation of cellular stress responses and suppression of proliferative programs following combination treatment.

To delineate broader pathways associated with this response, we performed Kyoto Encyclopedia of Genes and Genomes (KEGG) enrichment analysis. Both atovaquone and combination treatment suppressed glycolysis/gluconeogenesis and HIF-1 signaling pathways (Figure 4C and Figure S7), consistent with the metabolic and hypoxia-related effects observed experimentally. Combination treatment additionally showed repression of DNA replication and cell-cycle-associated pathways (Figure 4C), whereas atovaquone alone was associated with enrichment of pathways linked to steroid, cholesterol, and amino acid metabolism (Figure S7). Gene Set Variation Analysis (GVSA) showed enrichment of cholesterol homeostasis, p53, immune-related pathways (interferon gamma response, interferon alpha response, IL-6/JAK/STAT3 signaling, TNFα signaling via NFΚB, and IL2/STAT5 signaling) with atovaquone alone, alongside repression of hypoxia and cell-cycle programs (E2F, G2M). Addition of radiation resulted in stronger activation of the p53 pathway and pronounced repression of E2F and G2M checkpoint pathways (Figure S8). Collectively, these data indicate that atovaquone induces broad metabolic and transcriptional reprogramming in DIPG cells and, when combined with radiation, is associated with enhanced suppression of proliferative and cell-cycle-related pathways.

### 3.6. The Amorphous Solid Dispersion (ASD) Atovaquone Formulation Improves Brain Exposure and Enhances Radiation Response in an Orthotopic DIPG Model

**Figure 5:**
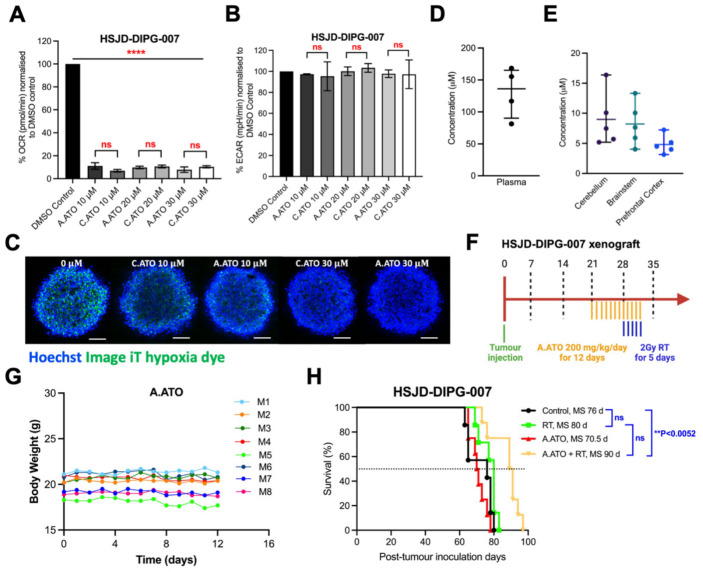
ASD atovaquone retains in vitro activity, achieves brain exposure, and enhances radiation response in an orthotopic DIPG model. (**A**,**B**) OCR and ECAR in HSJD-DIPG-007 cells treated with commercial atovaquone (C.ATO) or amorphous solid dispersion atovaquone (A.ATO), showing comparable effects of the two formulations on mitochondrial respiration and extracellular acidification. One-way ANOVA with Tukey’s multiple comparisons test was performed for all experiments; (ns = not significant, **** *p* < 0.0001). Data is presented as means ± SD of two independent experiments with three replicates in each; (**C**) Representative HSJD-DIPG-007 neurospheres stained with Image-iT Green Hypoxia Reagent after treatment with commercial or ASD atovaquone, showing similar reduction in hypoxia-associated fluorescence. Blue, Hoechst; green, hypoxia probe. Scale bar = 100 μm; (**D**) Plasma concentration of ASD atovaquone after repeated dosing in mice. (**E**) ASD atovaquone concentrations in cerebellum, brainstem, and prefrontal cortex (*n* = 5); (**F**) Experimental timeline for the HSJD-DIPG-007 orthotopic xenograft study; (**G**) Body weight during ASD atovaquone treatment (*n* = 8); (**H**) Kaplan-Meier survival curves showing prolonged survival with ASD atovaquone plus radiation compared with control, whereas ASD atovaquone or radiation alone did not significantly improve survival. Significance was calculated using the log-rank (Mantel-Cox) test (** *p* < 0.0052).

Because standard atovaquone has limited CNS penetration [19], we next evaluated if an amorphous solid dispersion (ASD) formulation would improve brain exposure [26]. We initially compared ASD atovaquone’s in vitro efficacy against commercially available atovaquone. There were no differences in the OCR and ECAR profiles between the two formulations (Figure 5A,B). Both formulations also decreased hypoxia to similar levels (Figure 5C). We then measured the plasma and brain concentrations achieved by ASD atovaquone in balb/c nude mice. After six consecutive days of ASD atovaquone dosing at 200 mg/kg, the average plasma concentration was 130.6 ± 39.40 μM (Figure 5D). ASD atovaquone concentrations in the cerebellum, brainstem, and prefrontal cortex were 9.0 ± 4.56 μM, 8.20 ± 3.62 μM, and 4.81 ± 1.55 μM, respectively (Figure 5E).

We next assessed whether ASD atovaquone enhanced radiation response in vivo using the HSJD-DIPG-007 orthotopic DIPG mouse model. Mice received ASD atovaquone (200 mg/kg/day) for 12 days, starting on day 21 (Figure 5F). Radiation (10 Gy total, 2 Gy/day) began on day 28, one hour after ASD atovaquone, to assess whether atovaquone could sensitize the DIPG tumor to radiation. ASD atovaquone treatment was not associated with overt toxicity as assessed by body weight over the treatment period (Figure 5G). Neither radiation nor ASD atovaquone alone prolonged the survival of mice, whereas the combination extended survival by 14 days compared to the control group (*p* value < 0.0052) (Figure 5H). These findings support ASD atovaquone as a formulation that achieves measurable brain exposure and enhances radiation response in an orthotopic DIPG model.

## 4. Discussion

This study provides preclinical evidence that atovaquone can moderately enhance radiation response in DIPG. Atovaquone rapidly suppressed mitochondrial respiration in DIPG cells, reduced hypoxia-associated readouts in neurosphere models, and enhanced radiosensitivity in vitro. Importantly, an ASD formulation achieved measurable brain exposure and, when combined with radiation, prolonged survival in an orthotopic DIPG model. Together, these findings support OXPHOS as a therapeutically relevant vulnerability in DIPG and identify atovaquone as a candidate radiosensitizer. Atovaquone’s established safety profile, short half-life, and tolerability in both children and adults support its suitability for clinical exploration [18].

Our findings strengthen the rationale for targeting oxidative metabolism in this disease. Atovaquone was a potent OXPHOS inhibitor in DIPG in vitro, suppressing mitochondrial metabolism at lower micromolar concentrations than phenformin or metformin [7], without reducing short-term viability. Moreover, the absence of an ECAR change suggests that DIPG cells may have limited metabolic flexibility following atovaquone. Additionally, the most direct mechanistic interpretation of our data is that atovaquone inhibits mitochondrial respiration through a block at complex III, consistent with prior reports in other tumor models [14,22].

Mitochondrial inhibition by atovaquone was accompanied by reduced hypoxia probe staining and lower HIF-1α expression in DIPG neurospheres, supporting hypoxia modification as one possible contributor to radiosensitization. However, radiosensitization was also observed in monolayer cultures at higher atovaquone concentrations, together with increased mitochondrial and cellular ROS, suggesting that oxidative stress may provide an additional mechanism under conditions where oxygen is more abundant. Our transcriptomic and metabolomic analyses further support broader treatment-associated reprogramming, including altered TCA-related metabolites and suppression of DNA replication and cell-cycle programs, although these findings should be viewed as exploratory rather than definitive mechanistic proof.

A major translational challenge for atovaquone in brain tumors is limited brain exposure. Here, the ASD formulation retained comparable in vitro biological activity to commercial atovaquone while achieving measurable plasma and brain concentrations in vivo, including in the brainstem. ASD atovaquone at 100 mg/kg has been previously shown to achieve concentrations of ~37.2 μM in the brain [26], whereas our study achieved ~10 μM brain level from ASD atovaquone dosed at 200 mg/kg. The difference in the findings might be explained by differences in the tissue processing approach between the studies. Unlike the previous study, we perfused brains with saline prior to excising the sections. Nevertheless, these brain levels exceed those with previously prepared nanosuspension and nanoemulsion atovaquone formulations that achieved around 0.90 and 1.36 μM atovaquone, respectively [27,28], indicating enhanced brain exposure. In the orthotopic HSJD-DIPG-007 model, neither radiation nor ASD atovaquone significantly prolonged survival, whereas the combination extended median survival by 14 days. This benefit was modest, and all animals ultimately succumbed to disease.

Several limitations should be acknowledged. Mechanistic conclusions remain partly inferential, as we did not directly test the requirement for hypoxia reduction, ROS induction, or cell-cycle suppression in vivo in mediating radiosensitization. The omics analyses and in vivo efficacy studies were each performed in a single DIPG model, and more detailed pharmacokinetic, pharmacodynamic, and toxicity studies will be needed to support translation of atovaquone or other mitochondrial metabolism inhibitors. Nonetheless, these data provide proof-of-concept support for atovaquone as a candidate radiosensitizer in DIPG and support further investigation of mitochondrial metabolism as a strategy to enhance radiotherapy response in DIPG brain tumors.

## 5. Conclusions

In summary, atovaquone enhanced radiosensitivity of DIPG models in vitro, and the BBB-penetrant ASD formulation significantly prolonged survival in the orthotopic DIPG model when combined with radiation. As radiotherapy remains the cornerstone of DIPG management, targeting mitochondrial metabolism represents a promising strategy to enhance radiosensitization and improve therapeutic outcomes. Given atovaquone’s safety profile, further optimization to enhance brain penetration could extend its utility to other brain tumors, including glioblastoma, ependymoma, and medulloblastoma. Clinical translation may be facilitated by biomarker-driven approaches and advanced imaging to guide patient selection and therapeutic response.

## Figures and Tables

**Table 1:** Top 10 differentially expressed genes unique to atovaquone + radiation vs control.

Atovaquone + Radiation vs. Control				
Gene Name	LogFC	LogCPM	FDR	Biological Implications
ANLN	−2.68	6.17	7.1 × 10^14^	Cytokinesis, cell division
EXO1	−2.45	5.60	1.35 × 10^13^	DNA repair pathways
MCM10	−2.56	5.37	1.35 × 10^13^	DNA replication
FEN1	−2.11	6.44	1.35 × 10^13^	DNA repair pathways
MCM4	−2.24	8.10	1.91 × 10^13^	DNA replication
NCAPH	−2.61	5.62	1.95 × 10^13^	Mitosis, chromosome segregation
CHAF1A	−2.22	6.30	1.95 × 10^13^	DNA replication and mitosis
KIF15	−2.41	5.04	2.22 × 10^13^	Mitosis
TICRR	−2.81	5.28	2.36 × 10^13^	DNA replication
WDR76	−2.02	5.32	4.01 × 10^13^	DNA damage sensing, chromatin regulation

## Data Availability

All the data generated by this study are presented in this manuscript in the main and Supplementary Figures. For additional inquiries, the corresponding authors can be contacted.

## Supplementary Materials

The following supporting information can be downloaded at: https://www.mdpi.com/article/10.3390/cancers18101553/s1, Supplementary Figure S1. % extracellular acidification rate (ECAR) of DIPG cell lines measured using Seahorse XFe24 Analyser following acute injection of atovaquone. Supplementary Figure S2. Effect of atovaquone on the viability on DIPG cell lines and hTERT IHA assessed using MTS cell proliferation assay at 72 hr treatment (* *p* < 0.05, **** *p* < 0.0001, vs. control). Supplementary Figure S3. mRNA levels of 11 mitochondrial complex III genes in DIPG/DMG clinical samples relative to non-tumor brain samples. Tumor samples included 10 brainstem glioma (BS) DIPG and 220 DMG-H3K27M samples. Non-tumor brain samples included 246 caudate (CAUD) and 202 hypothalamus (HYPO) samples. Statistical analysis was performed using one-way ANOVA with Tukey’s multiple comparisons test (* *p* < 0.05, ** *p* < 0.01, *** *p* < 0.001, **** *p* < 0.0001). Supplementary Figure S4. PCA scores plot of control, atovaquone, RT and atovaquone+RT groups. ATO, atovaquone; RT, radiation. Supplementary Figure S5. Top 10 differentially expressed genes unique to atovaquone+radiation vs control treatment. ATO, atovaquone; RT, radiation. Supplementary Figure S6. Top 10 differentially expressed genes unique to atovaquone vs control treatment. ATO, atovaquone; RT, radiation. Supplementary Figure S7. Transcriptomic alterations in HSJD-DIPG-007 cells treated with atovaquone. Dotplot analysis of KEGG enrichment showing the top altered pathways from atovaquone vs. control. Supplementary Figure S8. GVSA scores of pathways altered in HSJD-DIPG-007 following indicated treatments. Each dot per condition represents a replicate. ATO, atovaquone; RT, radiation. Supplementary Table S1. Top 10 differentially expressed genes unique to atovaquone vs. control treatment. Supplementary Table S2. Top 50 upregulated and 50 downregulated genes in HSJD-DIPG-007 cells following atovaquone treatment. Supplementary Table S3. Top 50 upregulated and 50 downregulated genes in HSJD-DIPG-007 cells following atovaquone+radiation treatment. File S1: Original western blots.

## Abbreviations

DIPG	Diffuse intrinsic pontine glioma
DMG	Diffuse Midline Glioma
HGG	High grade glioma
OXPHOS	Oxidative phosphorylation
ETC	Electron transport chain
ROS	Reactive oxygen species
OCR	Oxygen consumption rate
BBB	Blood brain barrier
ASD	Amorphous solid dispersions
CAUD	Caudate
HYPO	Hypothalamus
ECAR	Extracellular acidification rate
hTERT IHA	Immortalized human fetal astrocytes
HIF-1α	Hypoxia inducible factor 1α
PCA	Principle component analysis
TCA	Tricarboxylic acid cycle
DEGs	Differentially expressed genes
KEGG	Kyoto Encyclopedia of genes and genomes
GVSA	Gene set variation analysis
