# Supplementary material for: Atovaquone Targets Mitochondrial Metabolism and Enhances Radiosensitivity of Diffuse Intrinsic Pontine Glioma
Source: Cancers (Basel). 2026 May 11;18(10):1553. doi: 10.3390/cancers18101553 (PMC13204253; doi:10.3390/cancers18101553)

Please see attached raw western blot images from the blots presented in the manuscript. The blots in the manuscript are the same and have been cropped from the raw data as we only wanted to present time data for 0uM Atovaquone, 10uM atovaquone, 20uM atovaquone and 30uM atovaquone.

Thanks,
Dr Faiqa Mudassar

HSJD-DIPG-007

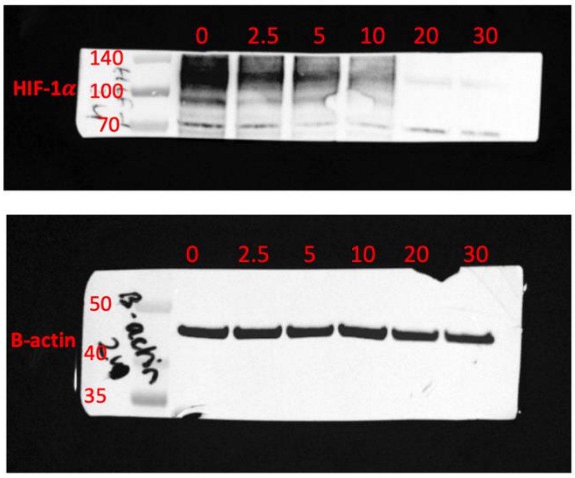

SU-DIPG-VI

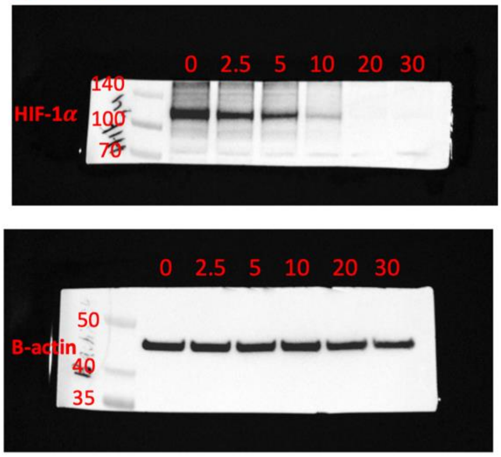

Supplement: Supplementary file 1 [file cancers-18-01553-s001.zip › cancers-4188578-File S1.pdf]
