# Supplementary material for: Atovaquone Targets Mitochondrial Metabolism and Enhances Radiosensitivity of Diffuse Intrinsic Pontine Glioma
Source: Cancers (Basel). 2026 May 11;18(10):1553. doi: 10.3390/cancers18101553 (PMC13204253; doi:10.3390/cancers18101553)
Supplement: Supplementary file 1 [file cancers-18-01553-s001.zip › cancers-4188578-supplementary.pdf]

Supplementary Figures

Supplementary figure S1. % extracellular acidification rate (ECAR) of DIPG cell lines measured using Seahorse XFe24 Analyser following acute injection of atovaquone.

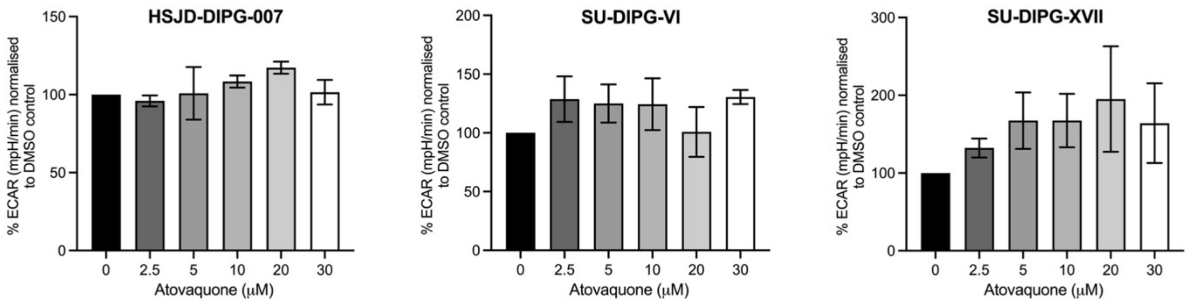

Supplementary figure S2. Effect of atovaquone on the viability on DIPG cell lines and hTERT IHA assessed using MTS cell proliferation assay at 72 hr treatment (*P < 0.05, ****P < 0.0001, vs control).

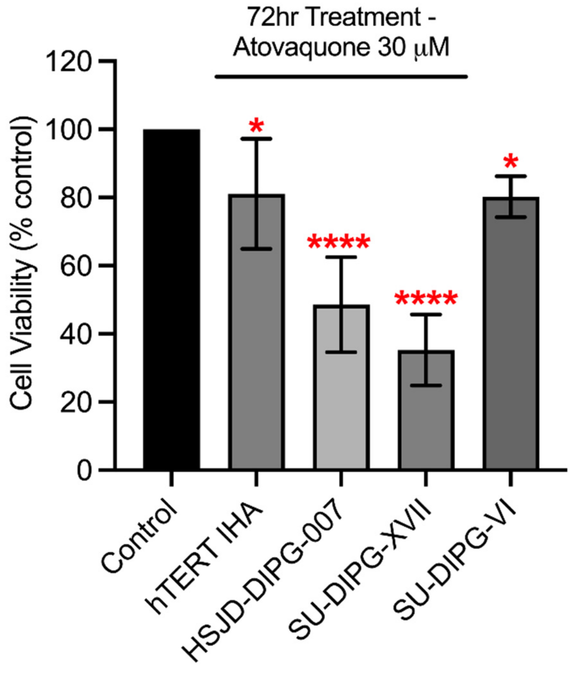

Supplementary figure S3. mRNA levels of 11 mitochondrial complex III genes in DIPG/DMG clinical samples relative to non-tumor brain samples. Tumor samples included 10 brainstem glioma (BS) DIPG and 220 DMG-H3K27M samples. Non-tumor brain samples included 246 caudate (CAUD) and 202 hypothalamus (HYPO) samples. Statistical analysis was performed using one-way ANOVA with Tukey's multiple comparisons test (*P < 0.05, **P < 0.01, ***P < 0.001, ****P < 0.0001).

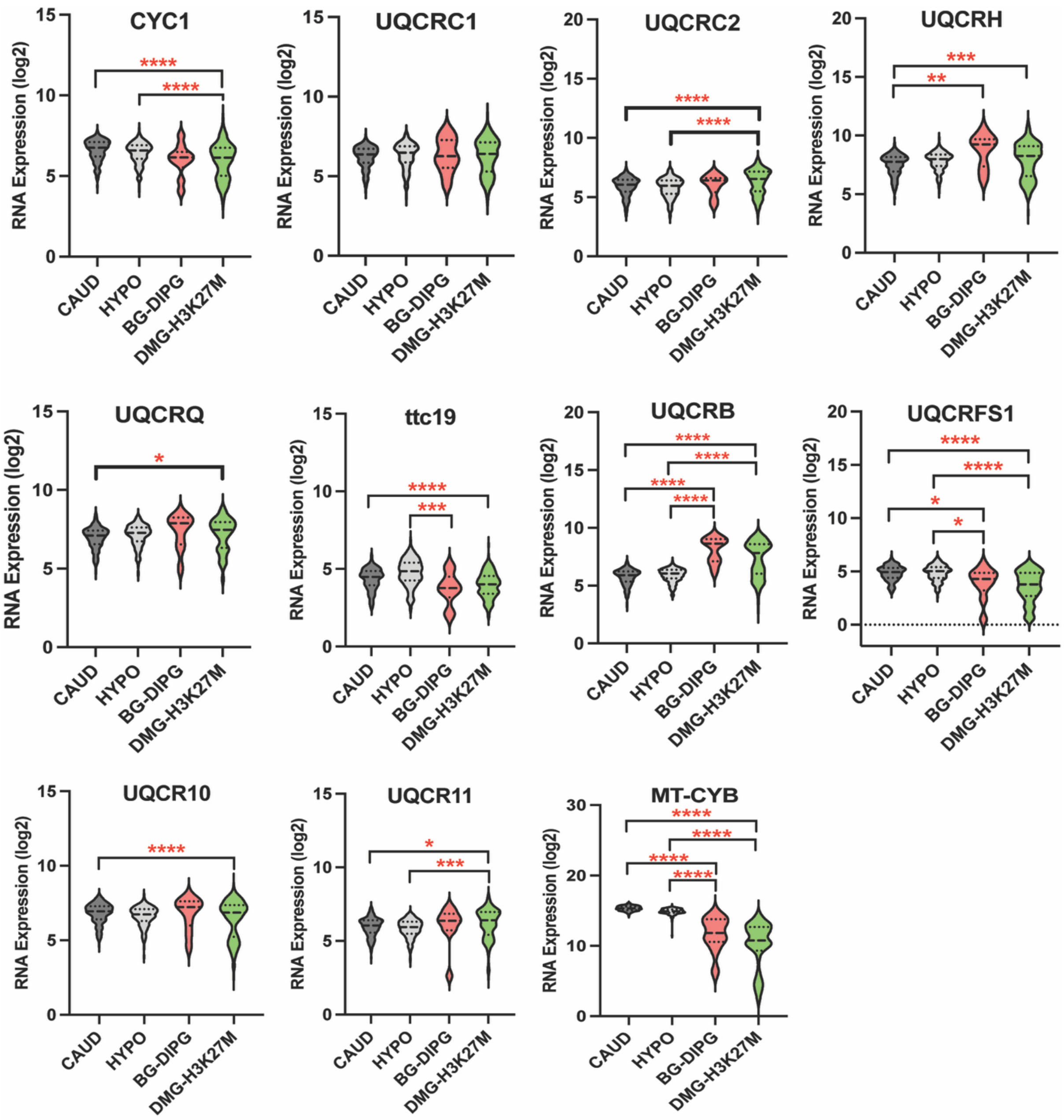

Supplementary figure S4. PCA scores plot of control, atovaquone, RT and atovaquone+RT groups. ATO, atovaquone; RT, radiation.

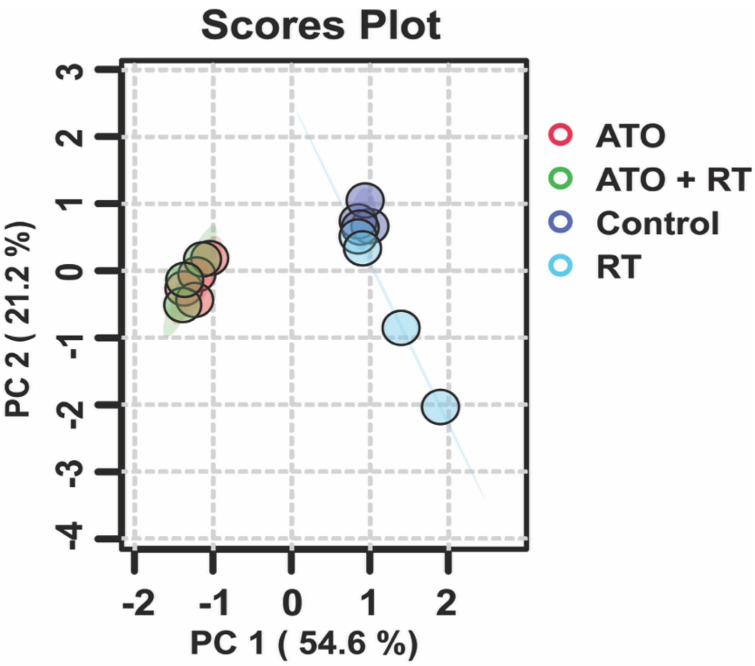

Supplementary figure S5. Top 10 differentially expressed genes unique to atovaquone+radiation vs control treatment. ATO, atovaquone; RT, radiation.

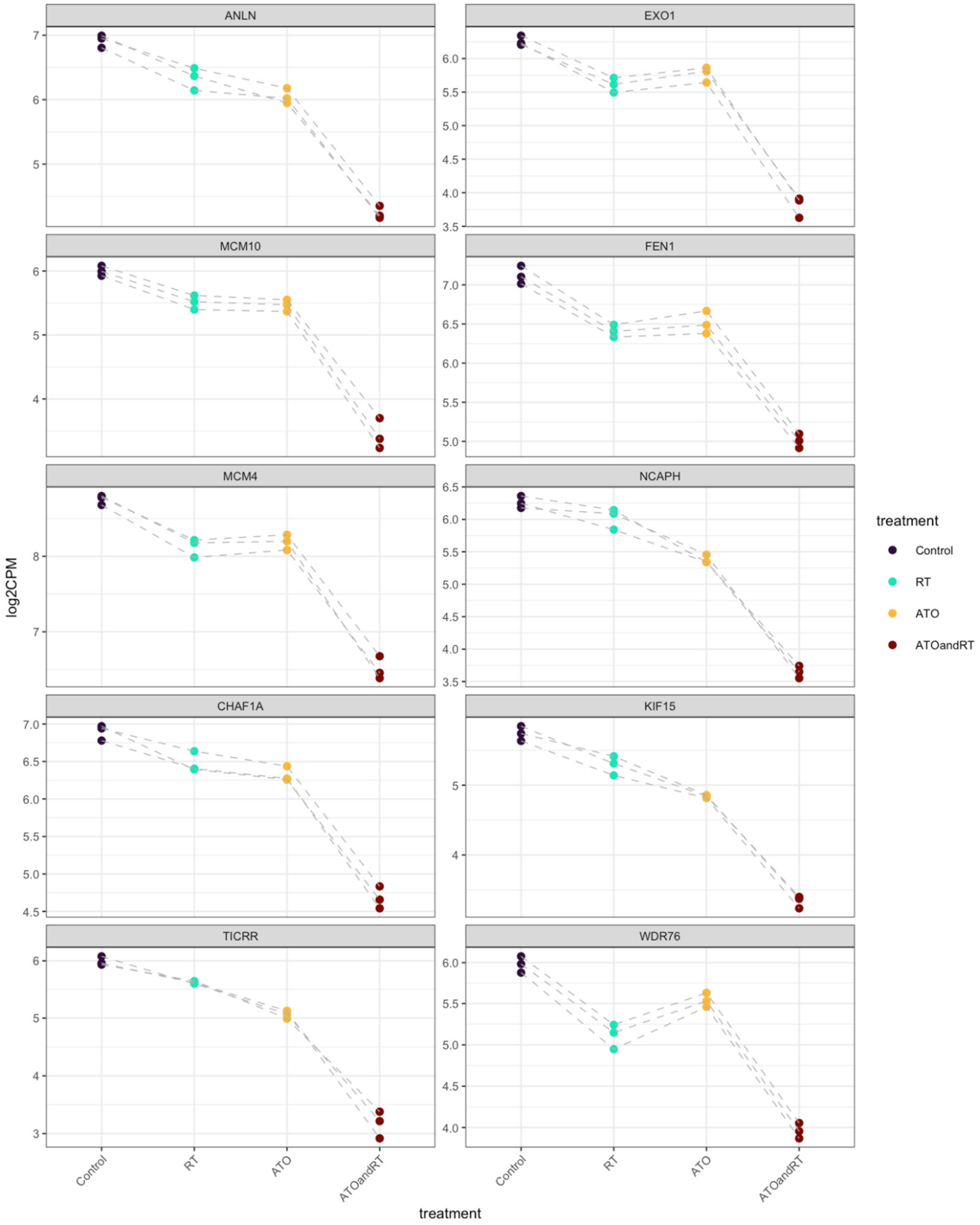

Supplementary figure S6. Top 10 differentially expressed genes unique to atovaquone vs control treatment. ATO, atovaquone; RT, radiation.

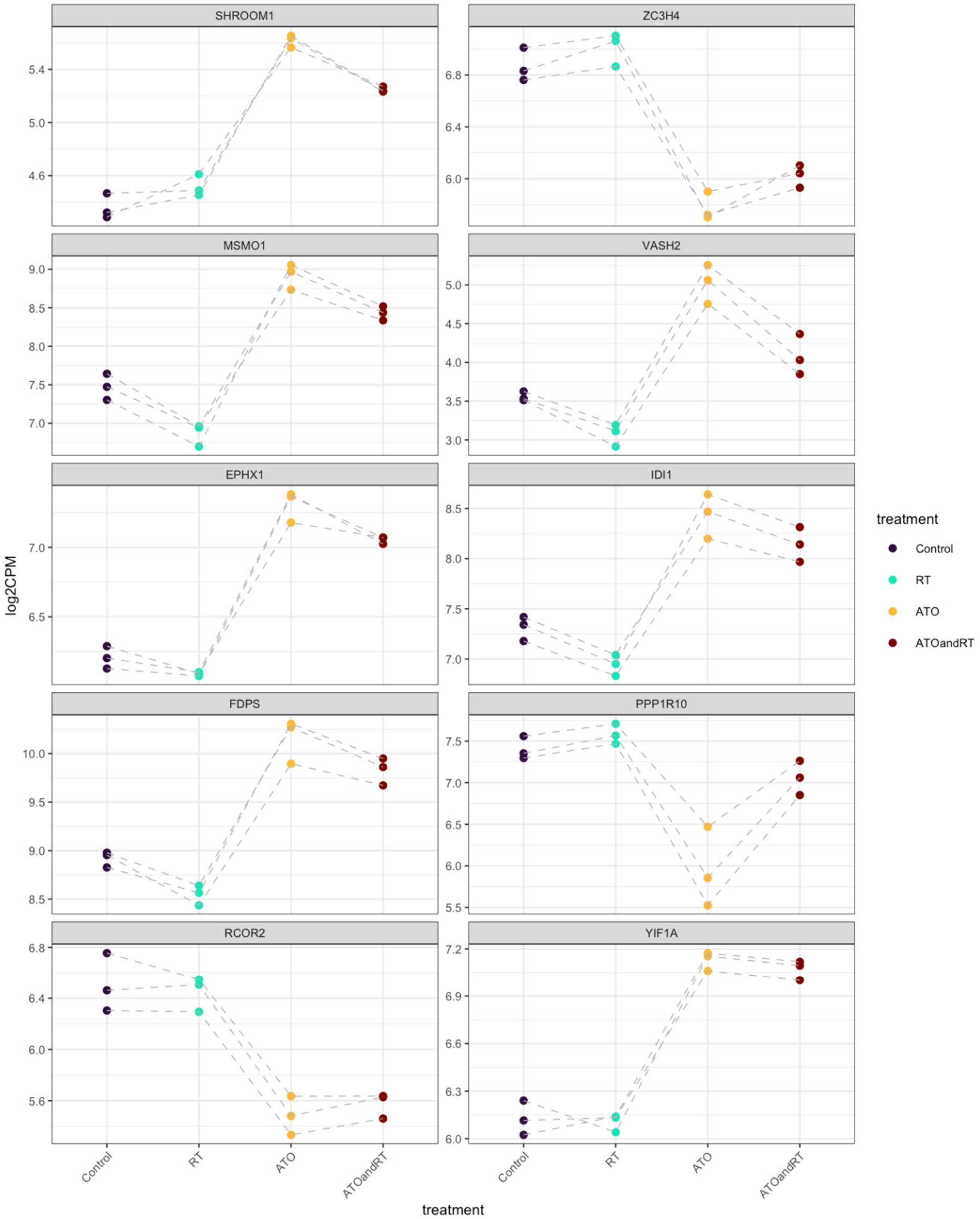

Supplementary figure S7. Transcriptomic alterations in HSJD-DIPG-007 cells treated with atovaquone. Dotplot analysis of KEGG enrichment showing the top altered pathways from atovaquone vs control.

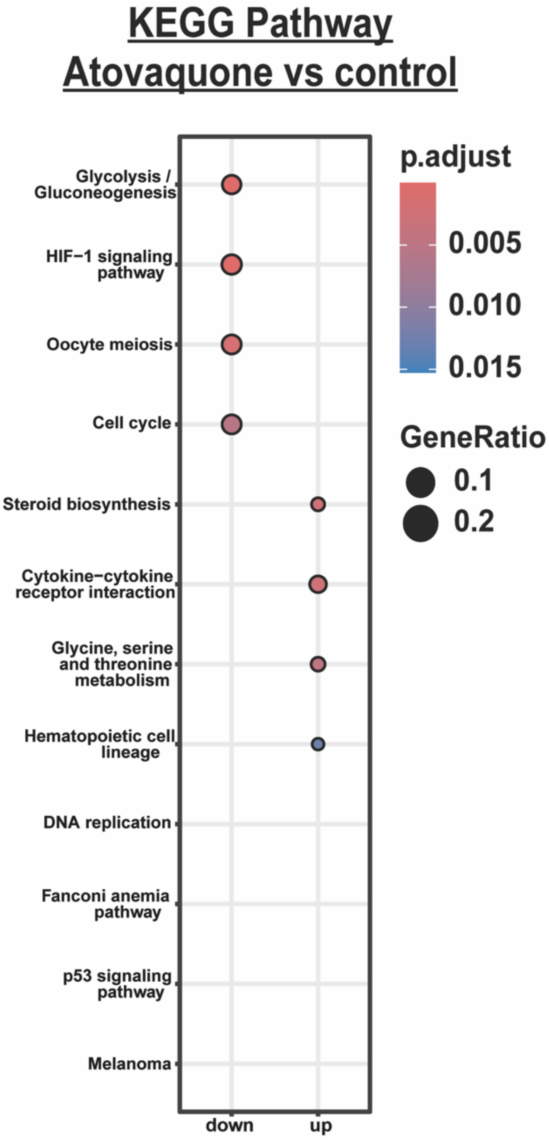

Supplementary figure S8. GVSA scores of pathways altered in HSJD-DIPG-007 following indicated treatments. Each dot per condition represents a replicate. ATO, atovaquone; RT, radiation.

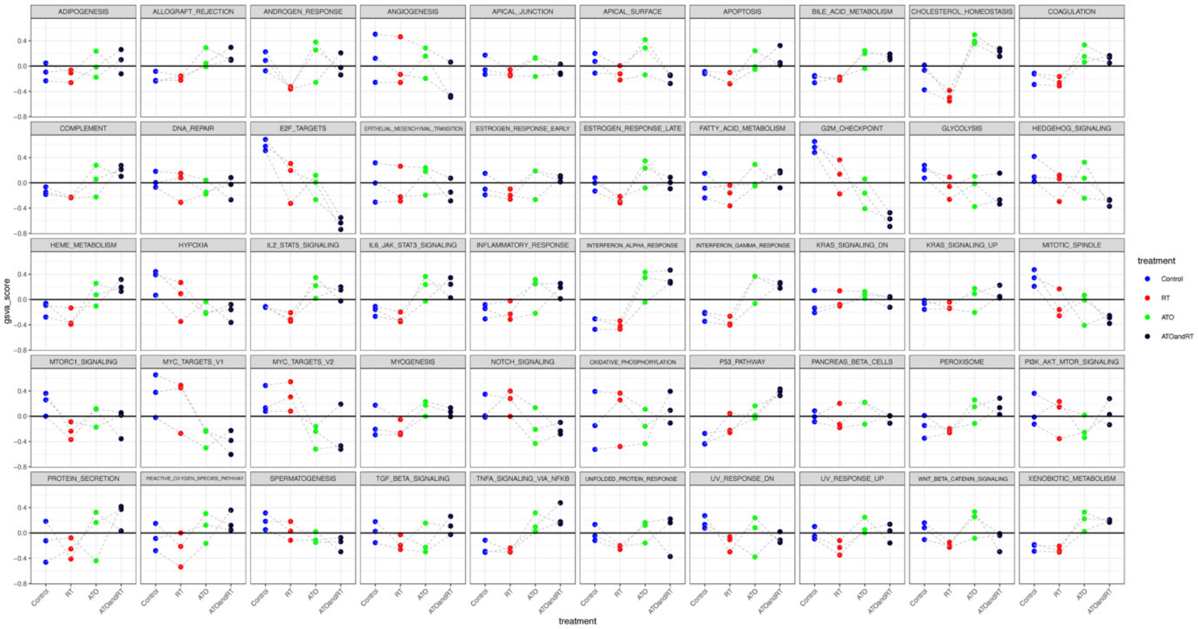

Supplementary Table S1. Top 10 differentially expressed genes unique to atovaquone vs control treatment.

Atovaquone vs control				
Gene Name	logFC	logCPM	FDR	Biological Implications
SHROOM1	1.26	5.03	2.21E-09	Cytokeletal organisation, cell migration
ZC3H4	-1.09	6.52	3.36E-09	RNA binding and gene expression regulation
MSMO1	1.45	8.14	3.46E-09	Cholesterol biosynthesis
VASH2	1.47	4.14	3.53E-09	Angiogenesis
EPHX1	1.1	6.76	3.82E-09	Detoxification and lipid metabolism
IDI1	1.12	7.84	4.29E-09	Isoprenoids and cholesterol biosynthesis
FDPS	1.24	9.51	7.23E-09	Isoprenoids and cholesterol biosynthesis
PPP1R10	-1.44	7.13	7.66E-09	Regulates DNA damage response
RCOR2	-1.03	6.09	8.88E-09	Epigenetic regulation, regulate DNA damage response
YIF1A	1	6.69	1.12E-08	ER-to-Golgi trafficking of proteins, autophagic responses

Supplementary Table S2. Top 50 upregulated and 50 downregulated genes in HSJD-DIPG-007 cells following atovaquone treatment.

Upregulated Genes				Downregulated Genes			
	Gene	Log2FC	P-value		Gene	Log2FC	P-value
1	FUT1	6.59	5.06E-11	1	ANGPTL4	-8.65	1.46E-18
2	PRODH	4.14	4.02E-05	2	PPDPFL	-8.48	2.72E-07
3	INHBE	3.93	3.54E-11	3	CA9	-5.62	3.33E-10
4	CHRNA9	3.89	1.14E-06	4	HNF1A	-5.39	1.28E-06
5	LINC00475	3.82	1.17E-05	5	STC1	-5.32	8.76E-15
6	FLRT1	3.76	9.08E-08	6	IGFBP3	-5.30	1.72E-17
7	GDF15	3.67	1.25E-16	7	PTGS2	-5.15	3.96E-15
8	MAP1LC3C	3.65	6.34E-11	8	ADM	-5.13	1.28E-16
9	CYBA	3.63	4.51E-08	9	MIR210HG	-5.03	2.83E-14
10	MYOM2	3.51	3.03E-11	10	AL109615.2	-4.87	1.62E-07
11	MIR7-3HG	3.48	7.86E-10	11	AC114803.1	-4.59	1.74E-10
12	TH	3.38	3.79E-07	12	NDRG1	-4.09	4.88E-13
13	GLS2	3.19	3.51E-04	13	AP002852.1	-3.92	1.51E-11
14	SLC6A9	3.18	2.07E-13	14	PTPRN	-3.91	9.53E-07
15	ASNS	3.16	7.32E-14	15	IGFBP5	-3.81	3.81E-14
16	AL139246.5	3.08	6.12E-09	16	TMEM45A	-3.77	6.87E-14
17	HHATL	3.07	1.17E-04	17	C4orf47	-3.68	8.36E-12
18	PLA2G4C	3.06	2.23E-11	18	DOK3	-3.64	7.67E-12
19	HES5	3.02	4.69E-07	19	SLAMF9	-3.60	6.07E-06
20	HAP1	2.96	4.09E-08	20	EGLN3	-3.59	3.70E-12
21	OSCAR	2.96	9.98E-07	21	APLN	-3.48	7.61E-07
22	DDIT3	2.78	7.22E-12	22	AK4	-3.33	1.40E-11
23	CACNG6	2.78	7.10E-08	23	AC093503.3	-3.32	6.36E-09
24	PPP1R14A	2.73	3.24E-04	24	HILPDA	-3.26	2.07E-14
25	IP6K3	2.72	5.52E-09	25	TCAF2	-3.23	4.11E-06
26	FABP3	2.64	6.76E-10	26	DDIT4L	-3.04	1.65E-07
27	FMNL1	2.62	5.12E-08	27	BNIP3	-3.04	2.54E-15
28	ITGA11	2.62	3.57E-08	28	LINC02783	-3.03	1.27E-06
29	TFRC	2.60	4.47E-13	29	FKBP9P1	-2.97	4.48E-11
30	SYTL1	2.54	1.19E-06	30	VWA1	-2.93	9.44E-11
31	ATF3	2.54	3.03E-10	31	GPR22	-2.93	7.19E-08
32	NUPR1	2.50	1.56E-13	32	ID2	-2.91	8.52E-12
33	AC004264.1	2.49	4.36E-07	33	LINC00683	-2.89	1.04E-06
34	TNXB	2.48	2.19E-08	34	PRTN3	-2.83	2.99E-07
35	IL11	2.47	6.81E-04	35	PGK1	-2.82	1.27E-14
36	GPAT3	2.45	2.66E-08	36	AC097534.2	-2.78	1.04E-10
37	PLIN4	2.44	1.37E-08	37	SLC2A1	-2.78	5.35E-13
38	NECTIN4	2.43	4.13E-07	38	ESPN	-2.77	4.17E-10
39	AC008897.3	2.43	1.09E-04	39	AMPD3	-2.73	2.12E-07
40	FAM114A1	2.43	1.08E-12	40	MYEOV	-2.69	7.83E-06
41	TRIB3	2.42	1.78E-12	41	LNP1	-2.68	1.62E-09

42	SCG2	2.40	1.13E-11	42	AL109615.3	-2.65	7.13E-08
43	CLDN4	2.39	6.77E-07	43	PFKFB4	-2.64	2.17E-11
44	IL3RA	2.37	3.18E-08	44	PDK1	-2.63	2.72E-15
45	IL21R	2.37	2.62E-07	45	AC078883.1	-2.62	1.59E-05
46	CSDC2	2.35	5.93E-11	46	HIC1	-2.60	5.90E-11
47	AC021739.3	2.34	2.79E-06	47	ENO2	-2.56	1.43E-11
48	PDIA2	2.34	1.46E-06	48	AC107021.2	-2.49	4.50E-05
49	CD70	2.34	1.09E-06	49	DARS-AS1	-2.48	1.25E-06
50	KCNN3	2.32	6.85E-10	50	AC022400.7	-2.46	4.02E-06

Supplementary Table S3. Top 50 upregulated and 50 downregulated genes in HSJD-DIPG-007 cells following atovaquone+radiation treatment.

Upregulated Genes				Downregulated Genes			
	Gene	Log2FC	P-value		Gene	Log2FC	P-value
1	FUT1	6.05	4.65E-10	1	PPDPFL	-8.50	1.97E-07
2	GDF15	5.76	1.42E-19	2	ANGPTL4	-8.49	1.17E-18
3	PRODH	5.55	6.77E-08	3	CA9	-6.90	9.02E-11
4	GLS2	5.26	3.52E-08	4	AL109615.2	-5.94	4.39E-08
5	HHATL	5.22	2.58E-09	5	IGFBP3	-5.38	1.20E-17
6	ABCA12	5.06	5.34E-07	6	AC114803.1	-5.19	5.90E-11
7	LINC00475	5.05	2.97E-08	7	PTGS2	-5.05	3.48E-15
8	WDR63	4.96	3.91E-08	8	MIR210HG	-5.04	2.26E-14
9	TRIM22	4.83	2.79E-14	9	STC1	-5.03	8.76E-15
10	NECTIN4	4.78	1.43E-12	10	ADM	-4.97	1.47E-16
11	IP6K3	4.69	1.37E-13	11	KIF20A	-4.81	7.18E-15
12	TFEC	4.61	4.71E-09	12	DDIT4L	-4.79	3.13E-09
13	ZNF423	4.55	3.48E-12	13	HNF1A	-4.71	1.58E-06
14	PLIN4	4.48	1.05E-13	14	NDRG1	-4.41	1.79E-13
15	MUC19	4.38	1.14E-08	15	LINC02783	-4.28	9.01E-08
16	FXYD2	4.37	1.49E-08	16	APLN	-4.20	1.71E-07
17	CDKN1A	4.33	9.96E-19	17	PLK1	-4.15	6.76E-14
18	CHRNA9	4.27	1.63E-07	18	AP001107.5	-3.98	1.71E-07
19	TMEM229B	4.07	3.24E-08	19	HRH3	-3.88	2.11E-09
20	MIR7-3HG	4.00	5.69E-11	20	SLAMF9	-3.88	2.90E-06
21	INHBE	3.98	2.62E-11	21	CENPA	-3.85	1.26E-12
22	PURPL	3.97	6.91E-11	22	IGFBP5	-3.68	5.96E-14
23	CD70	3.92	7.71E-11	23	CCNB1	-3.62	1.30E-13
24	PINCR	3.91	4.43E-08	24	DLGAP5	-3.60	2.94E-16
25	F11R	3.84	2.34E-08	25	HILPDA	-3.55	6.62E-15
26	AC004264.1	3.81	2.17E-10	26	GPR22	-3.54	1.07E-08
27	AL353138.1	3.76	8.19E-12	27	TMEM45A	-3.52	1.15E-13
28	PHLDA3	3.68	2.72E-17	28	AP002852.1	-3.50	3.53E-11
29	PPP1R14A	3.61	4.04E-06	29	EGLN3	-3.50	4.04E-12
30	IL11	3.57	2.93E-06	30	NEK2	-3.47	1.04E-12
31	PGF	3.56	4.42E-08	31	ASPM	-3.47	3.51E-15
32	AC068057.1	3.54	3.80E-07	32	CENPF	-3.46	5.91E-16
33	LNCTAM34A	3.41	5.75E-12	33	OLFM3	-3.41	6.14E-06
34	EBI3	3.37	1.81E-10	34	CDCA2	-3.40	2.58E-15
35	FLRT1	3.34	7.71E-07	35	HJURP	-3.40	4.24E-14
36	PLA2G4C	3.30	5.56E-12	36	CCNA2	-3.40	1.30E-14
37	EDA2R	3.28	1.87E-16	37	PIF1	-3.38	6.82E-13
38	GAS6-AS1	3.28	3.04E-07	38	MKI67	-3.32	6.32E-16
39	PLK2	3.28	5.95E-16	39	C4orf47	-3.31	1.95E-11
40	MAP1LC3C	3.22	5.54E-10	40	FAM83D	-3.27	6.51E-15
41	FDXR	3.20	1.41E-16	41	TCAF2	-3.25	2.96E-06

42	CYP4F2	3.15	1.15E-06	42	BUB1	-3.22	4.33E-16
43	PTCHD4	3.12	4.01E-17	43	CDC20	-3.20	1.03E-13
44	HAP1	3.11	1.58E-08	44	DOK3	-3.19	2.36E-11
45	OSCAR	3.10	4.21E-07	45	AL109615.3	-3.19	8.98E-09
46	GRIN2C	3.08	5.77E-09	46	AMPD3	-3.18	4.25E-08
47	LINC00346	3.05	1.37E-06	47	CDCA3	-3.18	3.55E-15
48	CSTA	3.04	1.84E-07	48	KIF18B	-3.17	1.94E-14
49	ROCK1P1	3.04	9.35E-09	49	AC097534.2	-3.17	2.01E-11
50	SLC6A9	2.98	6.55E-13	50	GTSE1	-3.17	4.89E-15
